# A maternal low-protein diet results in sex-specific differences in synaptophysin expression and milk fatty acid profiles in neonatal rats

**DOI:** 10.1017/jns.2024.46

**Published:** 2024-10-14

**Authors:** Paola C. Bello-Medina, Mauricio Díaz-Muñoz, Sandra Teresita Martín del Campo, Fermín Paul Pacheco-Moisés, Claudia Flores Miguel, Raquel Cobián Cervantes, Perla Belén García Solano, Mónica Navarro-Meza

**Affiliations:** 1 Facultad de Ciencias, Universidad del Tolima, Altos de Santa Helena, Ibagué, Tolima, Colombia; 2 Departamento de Neurobiología Celular y Molecular, Instituto de Neurobiología, Universidad Nacional Autónoma de México, Querétaro, México; 3 Escuela de Ingeniería y Ciencias, Tecnológico de Monterrey, Querétaro, México; 4 Food Engineering and Statistical Independent Consultant, Querétaro, México; 5 Departamento de Química, Universidad de Guadalajara, Guadalajara, Jalisco, México; 6 Laboratorio Clínica de Memoria y Neuronutrición, Departamento de Promoción, Preservación y Desarrollo de la Salud, Centro Universitario del Sur, Universidad de Guadalajara, Ciudad Guzmán, Jalisco, México; 7 Departamento de Ciencias Clínicas, División de Ciencias de Salud, Centro Universitario del Sur, Ciudad Guzmán, Jalisco, México

**Keywords:** Gestation, Hippocampus, Isocaloric low-protein diet (ILPD), Lactation, Metabolic markers, Synaptogenesis

## Abstract

The developmental origins of health and disease hypothesis have highlighted the link between early life environment and long-term health outcomes in offspring. For example, maternal protein restriction during pregnancy and lactation can result in adverse metabolic and cognitive outcomes in offspring postnatal. Hence, in the present study, we assess whether an isocaloric low-protein diet (ILPD) affects the fatty acid profile in breast milk, the hippocampal synaptophysin (Syn) ratio, and the oxidative stress markers in the neonatal stage of male and female offspring. The aim of this work was to assess the effect of an ILPD on the fatty acid profile in breast milk, quantified the hippocampal synaptophysin (Syn) ratio and oxidative stress markers in neonatal stage of male and female offspring. Female Wistar rats were fed with either a control diet or an ILPD during gestation to day 10 of lactation. Oxidative stress markers were assessed in serum and liver. All quantifications were done at postnatal day 10. The results showed: ILPD led to decreases of 38.5% and 17.4% in breast milk volume and polyunsaturated fatty acids content. Significant decreases of hippocampal Syn ratio in male offspring (decreases of 98% in hippocampal CA1 pyramidal and CA1 oriens, 83%, stratum pyramidal in CA3, 80%, stratum lucidum in CA3, and 81% stratum oriens in CA3). Male offspring showed an increase in pro-oxidant status in serum and liver. Thus, the data suggest that male offspring are more vulnerable than females to an ILPD during gestation and lactation.

## Graphical abstract

**Figure:**
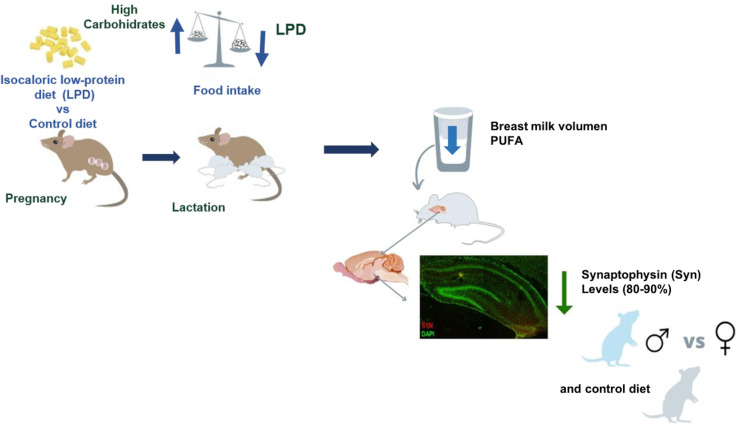

## Highlights

- Effects of an isocaloric low-protein diet (ILPD) consumption during pregnancy and lactation are related to a decrease in the hippocampal Syn ratio in male offspring. In contrast, female offspring were not affected.
- Effects of ILPD consumption during pregnancy and lactation are exclusively related to increased pro-oxidant status in the serum and liver of male offspring.
- The consumption of an ILPD during pregnancy affects the fatty acid profile of breast milk, particularly showing a reduction of polyunsaturated fatty acids.

In particular, the consumption of a low-protein diet (ILPD) during and after pregnancy has been associated with the development of metabolic and cognitive disorders, perinatal complications,^(1–3)^ and detrimental effects on body weight and blood pressure, as well as on the offspring metabolic and nutritional regulatory functions, such as the regulation of food intake.^(4,5)^ It’s known that developmental origins of health and disease (DOHaD) in early life environments can impact the risk of chronic diseases from childhood to adulthood and the mechanisms involved.^(6)^ Obesity has also been related to intrauterine and postnatal growth restriction, due to a deficiency in key specific amino acids that are important for metabolism and development.^(7)^ The number of cases of chronic diseases during pregnancy, such as diabetes and gestational obesity, preeclampsia, hypertension, and macrosomia, has increased during the last decades,^(8,9)^ and pregnant human mothers are advised to restrict their consumption of high-calorie foods, which have a particularly low nutritional value.^(10)^ In experimental murine models, the consumption of an ILPD has been related to an increase in visceral adiposity^(11)^ and to a significant increase in liver fat microvesicles and macrovesicles.^(12)^ It has been recognised that lactation is essential to prevent chronic diseases in the postnatal stage; therefore, the consumption of foods with adequate macronutrients and fatty acids should be promoted in the reproductive age.^(13)^ However, further research is needed to identify other determinants of the fatty acid profile of breast milk, as well as its potential effects on the cognitive function and postnatal development of offspring. One of the neural structures most susceptible to cellular stress and damage is the hippocampus, which plays a vital role in the processes of learning, spatial navigation, and memory consolidation. Previous studies have reported behavioural and cognitive alterations,^(14)^ as well as morphological and functional effects in offspring of mothers that consumed an ILPD diet during pregnancy and lactation.^(15)^ However, such alterations have not been further studied comparing male and female neonates. One of the markers implicated in the formation of cerebral synapses is the presence of synaptophysin (Syn). Syn is involved in structural plasticity related to learning and memory by regulating synaptic transmission in neuronal circuits.^(16)^ Intrauterine growth restriction in rats has been shown to result in delayed cortical synaptogenesis, myelination, and oxidative injury,^(17)^ although sex-specific differences were not examined. The present research focuses on evaluating the effects of consuming an ILPD during pregnancy on the fatty acid profile of breast milk; metabolic and oxidative stress markers in serum and liver, as well as hippocampal Syn levels in offspring at postnatal day 10 (PND10) in the male and female offspring. During this early neonatal stage, the hippocampus undergoes rapid development by morphogenesis and synaptogenesis of neuronal cells.

## Materials and methods

The procedures were carried out following the Official Mexican Standard NOM-062-ZOO-1999. This research was reviewed and authorised by the Ethics Committee of the Neurobiology Institute, UNAM (México) (approval ID 081. A).

### Procedure

Rats were individually held in cages (21 × 23.5 × 38 cm), under controlled 12 h dark/light conditions with lights on at 07:00 h. During 21 d of gestation (G) and 10 d of lactation (L) the food intake and the body weights of mothers were recorded regularly.

*Ad-libitum* food and water intake was quantified before pregnancy, during pregnancy, and during lactation. Body weight was measured with a precision scale (*A&D Weighing series GF-3000 scale, South Korea*). The control diet (AIN 93G *Test diet,* 63/18%) contained 63.2% carbohydrates, 7.1% fat, and 18% protein, and the experimental diet (ILPD) (AIN 93G *Test diet,* 77/6%) contained 77.3% carbohydrates, 7.1% fat, and 6% protein (Table 1). The proestrus-oestrus stage was identified by a vaginal smear. Once this phase was observed, females were placed with a mature male rat, for mating; fertilisation, determined by a vaginal smear, denoted gestation day 0 (G0).

**Table 1.** Nutritional content of the control and experimental diet

	Control		Experimental
Energía (Kcal/g)^2^	kcal (%)	Energía (kcal/g)^2^	kcal (%)
Proteína	0.731 (18.3)	Proteína	0.240 (6)
Carbohidratos	2.528 (64.9)	Carbohidratos	3.090 (77.8)
Grasas	0.637 (16.4)	Grasas	0.640 (6.1)
Arginine %	0.70	Arginine %	0.21
Histidine %	0.52	Histidine %	0.15
Isoleucine %	0.96	Isoleucine %	0.28
Leucine %	1.73	Leucine %	0.51
Lysine %	1.45	Lysine %	0.43
Methionine %	0.52	Methionine %	0.15
Cystine %	0.27	Cystine %	0.32
Phenylalanine %	0.96	Phenylalanine %	0.28
Tyrosine %	1.01	Tyrosine %	0.38
Threonine %	0.77	Threonine %	0.23
Tryptophan %	0.22	Tryptophan %	0.07
Valine %	1.14	Valine %	0.34
Alanine %	0.55	Alanine %	0.16
Aspartic Acid %	1.29	Aspartic Acid %	0.38
Glutamic Acid %	4.08	Glutamic Acid %	1.21
Glycine %	0.39	Glycine %	0.11
Proline %	2.36	Proline %	0.70
Serine %	1.10	Serine %	0.33
Taurine %	0.000	Taurine %	0.00
Cholesterol, ppm	0	Cholesterol, ppm	0
Linoleic Acid %	3.58	Linoleic Acid %	3.58
Linolenic Acid %	0.55	Linolenic Acid %	0.55
Arachidonic Acid %	0.00	Arachidonic Acid %	0.00
Omega 3 Fatty Acids%	0.55	Omega 3 Fatty Acids%	0.55
Total Saturated Fatty Acids %	1.05	Total Saturated Fatty Acids %	1.05
Polynsaturated Fatty Acids %	1.54	Polynsaturated Fatty Acids %	1.54
Fibre %	3.78	Fibre %	3.78
Calcium %	0.51	Calcium %	0.51
Phosphorus %	0.32	Phosphorus %	0.32
Potassium%	0.36	Potassium%	0.36
Magnesium %	0.05	Magnesium %	0.05
Sodium %	0.13	Sodium %	0.13
Chloride %	0.22	Chloride %	0.22
Flurine, ppm	1.0	Flurine, ppm	1.0
Iron, ppm	40	Iron, ppm	40
Zinc, ppm	35	Zinc, ppm	35
Manganese, ppm	11	Manganese, ppm	11
Copper, ppm	6	Copper, ppm	6
Cobalt, ppm	00	Cobalt, ppm	00
Iodine, ppm	0.21	Iodine, ppm	0.21
Chromium, ppm	1.0	Chromium, ppm	1.0
Molybdenum, ppm	0.14	Molybdenum, ppm	0.14
Selenium, ppm	0.24	Selenium, ppm	0.24
Vitamin A, IU/g	4	Vitamin A, IU/g	4
Vitamin D-3, IU/g	1.0	Vitamin D-3, IU/g	1.0
Vitamin E, IU/kg	81.6	Vitamin E, IU/kg	81.6
Vitamin K,ppm	0.75	Vitamin K,ppm	0.75
Thiamine, ppm	4.8	Thiamine, ppm	4.8

### Control and experimental group

Wistar rats weighing 250–300 g were randomly organised into two groups: Pregnant rats fed with a control diet (Control, n = 3) and pregnant rats fed with an ILPD (Experimental, n = 3). The offspring number per group was quantified for each experiment. From each mother in the control or experimental group, one female or male offspring was selected for immunohistochemical analysis to detect Syn in the hippocampus on postnatal day 10 (PND10), the final sample size was six offspring per group (n=6). For analysis of fatty acids in maternal milk, three male or female offspring were selected per group (n = 6). For the evaluation of pro-oxidant markers, we selected blood and liver samples from four male and female offspring per group (n = 4).

The litter effect was tested based on a nonlinear mixed model approach by applying the *R-package ‘nlme’* to our data, with the litter variable as a random effect.^(18)^ We verified that there is no litter effect through *SPSS version 19.0 software* as follows: we calculated the descriptive statistics and frequency distribution, and adjustments were made for the weight of the pups at PND10. The 12 pups were variably selected by the litters that include the control group and the experimental group, being verified and calculated through multivariate analysis adjusted by litter effect of the pups. Results are shown in Appendix 1.

### Collection of milk samples

At PND10 the offspring were kept in a room at 25–28°C during the milk extraction procedure. The milk was collected with a vacuum system and stored at –20°C for subsequent analysis. The offspring were then returned to their mothers to be fed for 1 h and were subsequently weighed. This protocol was retrieved from.^(19)^

### Analysis of fatty acids in maternal milk

Milk lipids were extracted by Folch’s method^(20)^ as follows: 1 ml of hexane (*HPLC grade, KARAL, México*) was added to the samples, and 50 mg of the retrieved fat was taken and mixed vigorously. Next, 100 μl of 5N sodium methoxide (Sigma-Aldrich, USA) was added; the samples were incubated at 50°C for 5 min. Then 5 ml of distilled water and 0.1 ml of glacial acetic acid were added and gently stirred in. Fatty acid methyl esters were retrieved by double extraction using 3 ml of hexane. The organic extracts were dried over anhydrous sodium sulfate and placed in closed vials for further analysis by gas chromatography. Fatty acid methyl esters were separated on an HP-88 column (88% Cyanopropy) aryl-polysiloxane, 100 m × 0.250 mm × 0.20 μm (Agilent Technologies, Ca. USA) on a 7890 A GC gas chromatograph (Agilent Technologies, Ca. USA), together with a 5975C mass selective detector (Agilent Technologies Ca. USA), and a CTC CombiPAL autosampler (Zwingen, Switzerland). The injection port and interface temperature were stabilised at 250°C. One µl was injected for each sample, and a split ratio of 2:1 was set. Helium was used as carrier gas at a flow rate of 1 ml/min. The oven was programmed to start at 50°C, and immediately increased to 85°C at 2.5°C/min. Then, it was increased from 10°C/min to 170°C for 20 min. Finally, the temperature was increased at 10°C/min until reaching 250°C/ 25 min. The mass selective detector integrated an electron impact (EI) system, and spectra were obtained at 70 eV and 1.6 scans/s; the acquisition mass range (m/z) was 30–350. The lipid profile was identified using the *MSD Chem Station* E.02.00.493 software (Agilent Technologies, USA), and compared with the National Institute of Standards and Technology database. *Fatty Acids Methyl Esters* were quantified with a calibration curve of reference standards of saturated, monounsaturated, and polyunsaturated fatty acid methyl esters (Sigma-Aldrich, USA).^(21,22)^

### Brain sampling and slices

Offspring were euthanized due to exposure to an environment with CO_2_. The anaesthesia was applied until no vital signs were detected. The brains were obtained, frozen in a mixture of dry ice and 100% ethanol (v/v) for 5 min and stored at –80°C. Blocks containing six brains of each experimental condition were made. Each brain section contained the hippocampus (Bregma –2.52 to –4.56 mm).^(23)^ Slices 20 µm thick were obtained on a Leica cryostat. Hippocampal slices were placed on gelatinised slides and stored at –80°C.

### Immunohistochemical detection of synaptophysin

**Fig. 1.**
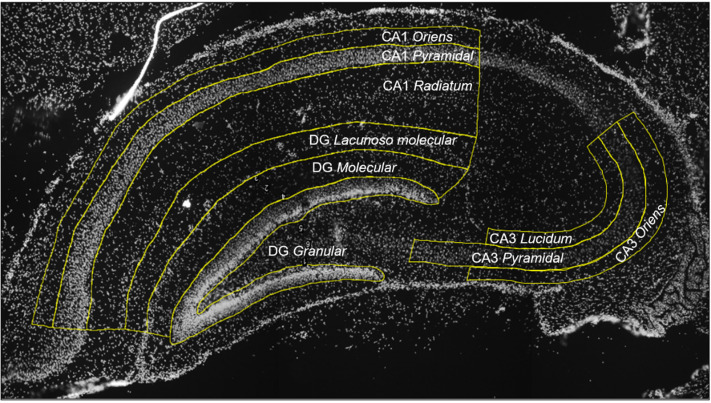
This image shows the drawings that defined the different hippocampal areas considered in this study (CA1, CA3), as well as the dentate gyrus on the dorsal hippocampus in offspring at PND10.

The immunohistochemical technique was performed to detect Syn protein. Hippocampal sections were fixed in 2% buffered paraformaldehyde, and washed with TBS (*trizma buffer*) each for 10 min. Endogenous peroxidases were blocked with 30% H_2_O_2_ for 20 min, and washed with TBS for 10 min. Subsequently, TSA (PerkinElmer, MA USA) was added for 60 min. An overnight incubation at 4°C with mouse anti-Syn (1:500, Sigma Cat# S5768) was later performed. Subsequently, were washed with TBS for 10 min. Biotinylated goat-anti-mouse incubation (1:500, Vector Cat# BA-9200) was performed for 2 h at room temperature. Washes were performed with TBS for 10 min. A+B (avidin + biotin, Vector Labs, CA USA) incubation was performed for 1 h, and washed with TBS for 10 min. Incubation with TSA system FITC (1:1000, Perkin Elmer kit, MA USA) was performed for 30 min. 4′,6-diamidino-2-phenylindole (DAPI) (1:5000) counterstaining was performed to visualise the cell nuclei. VectaShield mounting medium was applied, and coverslips were placed over the slides. Six hippocampal mosaic images were obtained (Bregma –2.92 to –3.96 mm^(23)^ with the 25x/0.8 NA objective lens and the MosaiX module for the Apotome system (Zeiss). Analysis of Syn presence was performed using the ImageJ software, the procedure was carried out as described previously.^(24–27)^ The results were expressed as hippocampal area ratio (the area that is occupied by Syn in the total area of *pyramidal, oriens*, or *radiatum* stratum of CA1, *pyramidal, oriens,* or *lucidum* stratum of CA3, *granular, molecular,* or *lacunosum molecular* stratum of the dentate gyrus (DG) (Fig. 1).

### Evaluation of pro-oxidant markers

Blood samples (n = 4 per group) were centrifuged at 2000×g for 10 min at room temperature to obtain serum, Lipid peroxidation products (malondialdehyde plus 4-hydroxyalkenals) in serum were quantified using the reagent N-methyl-2-phenylindole as reported in previous studies.^(28)^ Nitric oxide metabolites in serum were determined by adding 100 μl of vanadium chloride at a concentration of 8 mg/ml to 50 μl of serum to reduce nitrites to nitrates. Griess reagent (comprising 50 μl of 2 % sulfanilamide, and 50 μl of 0.1% N-(1-naphthyl) ethylenediamine dihydrochloride) was then added. The samples were incubated for 30 minutes at 37°C, and the absorbance was read at 540 nm. A standard curve (0–150 μmol) of sodium nitrite was established.^(29)^

Liver from offspring (n = 4 per group) was homogenised 1:10 in ice-cold 50 mM tris (hydroxymethyl) aminomethane buffer (pH 7.4) with a Teflon-on-glass Potter-Elvehjem homogeniser. Homogenates were centrifuged at 3000 ×g for 20 min at 4°C. The supernatant was collected and immediately assayed for catalase activity and total antioxidant capacity. Catalase was assessed with 50 μl of serum mixed with 0.3 ml of reaction medium (65 mM hydrogen peroxide in 60 mM phosphate buffer, pH = 7.4), and incubated at 37°C for 2 min. The reaction was stopped with 1 ml of 32.4 mM ammonium molybdate. The absorbance of the samples was recorded at 374 nm to quantify the remaining hydrogen peroxide in the reaction. To exclude the interference of proteins and other compounds, a series of blanks with plasma and without substrate were used. The substrate solution was prepared immediately before use and was standardised using a molar extinction coefficient of 43.6 M^–1^ cm^–1^ at 240 nm.^(30)^ For total antioxidant capacity, the method used was based on the measurement of Cu(I)-neocuproine chelate absorbance, formed because of the Cu^2+^ reduction due to the antioxidants present in the samples. The absorbance was recorded at 450 nm and referred to as a standard curve of Trolox.^(31)^

### Statistical analysis

The Kolmogorov-Smirnov test was performed to evaluate the parametric assumption of normality. For parameters of food, water consumption, and body weight, we used *t*-student and one-way ANOVA tests. For Syn analysis, a one-way and two-way ANOVA was applied, where factor 1 was diet (control or experimental), and factor 2 was sex (female or male) for each dorsal hippocampal area (*pyramidal, oriens*, or *radiatum* stratum of CA1, *pyramidal, oriens,* or *lucidum* stratum of CA3, *granular, molecular,* or *lacunosum molecular* stratum of DG). The Bonferroni post hoc test was used when appropriate; *P < 0.05* was considered statistically significant. The litter effect was tested based on a nonlinear mixed model approach by applying the R-package ‘nlme’ to our data, with the litter variable as a random effect.^(18)^

## Results

### Maternal body weight and food/water intake in lactation

**Fig. 2.**
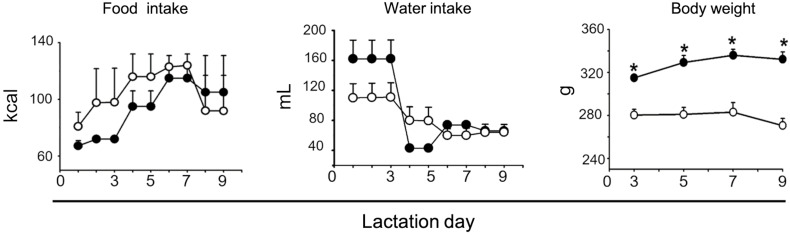
Food and water intake, and body weight in lactation; control group (black circles), and experimental group (isocaloric low-protein diet; white circles). (A) Food intake (kcal), (B) Water intake, (C) Body weight. Data are shown as mean ± standard error, *t*-test *P < 0.05, n = 3 rats per group.

No significant differences in food and water intake were observed in mother rats during lactation when comparing both the control and the ILPD groups (Fig. 2a and b). Body weight decreased significantly during lactation in the ILPD group; from day 3 to 9, the decrease was between 14% and 24%, when compared to the mothers fed with the control diet.

Figure 2c shows the weight gain; the control group weighed on average 332.3 ± 5.3 g at day 9, whereas the ILPD group weighed 270.6 ± 6.4 g. The experimental diet promoted a 19% reduction in body weight. Thirteen pups were born to mothers fed with a control diet 13, and 13 were born to mothers fed with an ILPD; an average of 8 males and 5 females’ offspring were registered in the control group, while an average of 6 males and 7 females’ offspring were registered in the experimental group.

### Synaptophysin area ratio in offspring dorsal hippocampus at postnatal stage 10

**Fig. 3.**
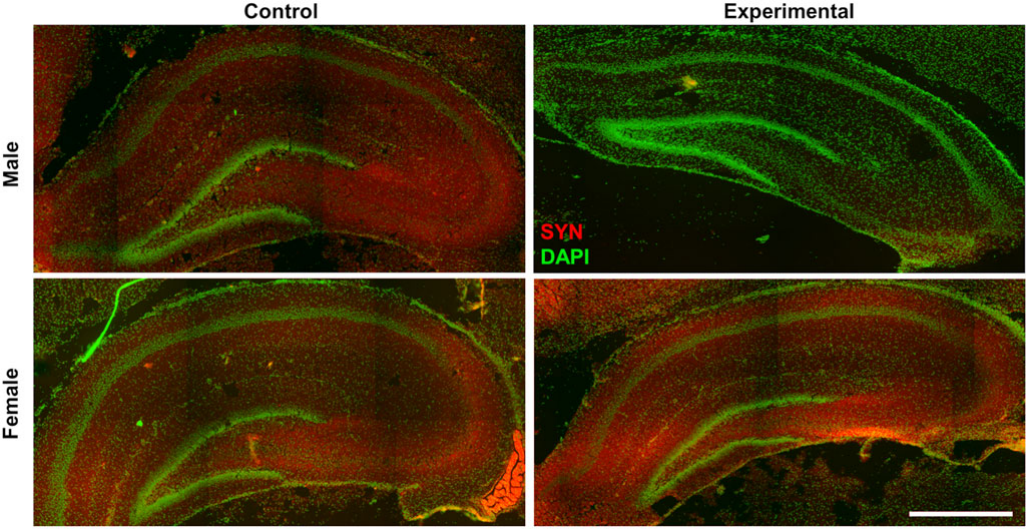
Representative images of immunohistochemical detection of synaptophysin (Syn, red) and nuclear staining (DAPI, green) in the dorsal hippocampus, in male and female offspring at PND10 that were fed with a low-protein diet (experimental, isocaloric low-protein diet) or control diet during gestation and lactation. Bar scale 500 μm.

**Fig. 4.**
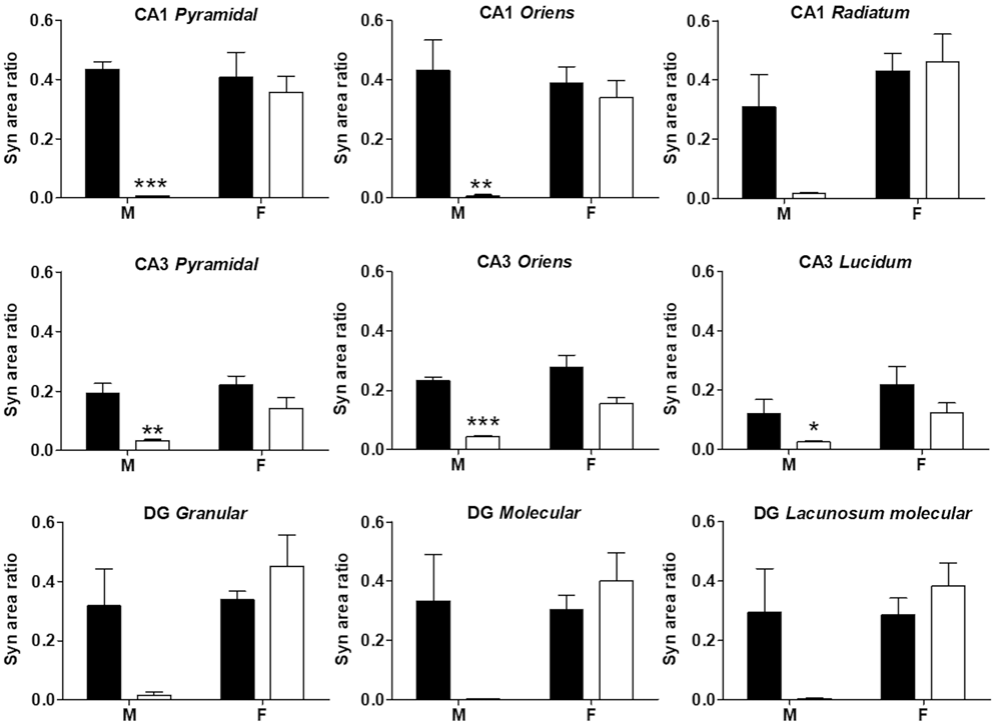
Effect of an isocaloric low-protein diet (ILPD) in gestation and lactation on synaptogenesis observed in CA1, CA3, and DG strata of dorsal hippocampus of male and female offspring at PND10 that were fed with control (black bar) or experimental diets (ILPD, white bar). Graphs show the mean ± standard error of the ratio of the area that is occupied for synaptophysin (Syn) in the pyramidal, *oriens*, and *radiatum* strata of CA1; *pyramidal*, *oriens*, and *lucidum* strata of CA3; *granular*, *molecular*, and *lacunosum molecular* strata of DG from the dorsal hippocampus. Data are shown as mean ± standard error. Two-way ANOVA * P < 0.05, ** P < 0.01, *** P < 0.0001, n = 6 rats per group.

ILPD consumption during gestation and lactation promoted a drastic reduction in the Syn area ratio in CA1 of the dorsal hippocampus-only male offspring at PND10. In comparison to the control group, a decrease of 98.6%, 98.3%, and 94.8% were respectively observed in *pyramidal* (diet F(1,8) = 21.60, P < 0.01; sex F(1,8) = 9.92, P < 0.05; interaction F(1,8) = 13.55, P < 0.01), *oriens* (diet F(1.8) = 13.43; P = 0.0064; sex F(1.8) =4.960; P = 0.056; interaction F(1,8) = 8.397; P = 0.0200), and *radiatum* stratum (diet F(1,8) = 2.870, P = 0.1287; sex (F(1,8) = 13.35; P = 0.0065), interaction F(1,8) = 4.337; P = 0.0708) in the Syn area ratio. On the other hand, no effect was observed in female offspring fed with an ILPD (Figs. 3 and 4). Similarly, a decrease in the Syn area ratio was observed in CA3 of the dorsal hippocampus at PND10 in male offspring fed with the ILPD diet during gestation and lactation (P < 0.05); In *pyramidal* (diet F(1,8) = 17.46, P = 0.0031; sex F(1,8) = 5.793, P = 0.0427; interaction F(1,8) = 1.909, P = 0.2044), *oriens* (diet (F(1,8) = 5.283, P = 0.0506); sex (F(1,8) = 5.621, P = 0.0452); interaction (F(1,8) = 0.00003158, P = 0.9986), and *lucidum* stratum (diet F(1,8) = 42.57, P = 0.0002; sex (F(1,8) = 10.67, P = 0.0114); interaction F(1,8) = 2.053, P = 0.1898) decreases of 82.8%, 81.0%, and 80.0 % respectively were observed, compared to the control group. Again, no effect was observed in female offspring fed with an ILPD (Figs. 3 and 4). In the same direction, in the dentate gyrus of the dorsal hippocampus, a non-significant decrease in the Syn area ratio in male offspring fed with an ILPD was also observed in the *granular* (diet F(1,8) = 1.28, P = 0.29; sex (F(1,8) = 7.53, P = 0.025); interaction F(1,8) = 6.16, P = 0.038), *molecular* (diet F(1,8) = 1.54, P = 0.25; sex (F(1,8) = 3.74, P = 0.089); interaction F(1,8) = 5.00, P = 0.06), and *lacunosum molecular stratum* (diet F(1,8) = 1.18, P = 0.31; sex (F(1,8) = 4.37, P = 0.07); interaction F(1,8) = 4.73, P = 0.06), compared to the control group. No effect was observed in female offspring fed with ILPD (Figs. 3 and 4).

### Pro-oxidant markers in offspring

**Fig. 5.**
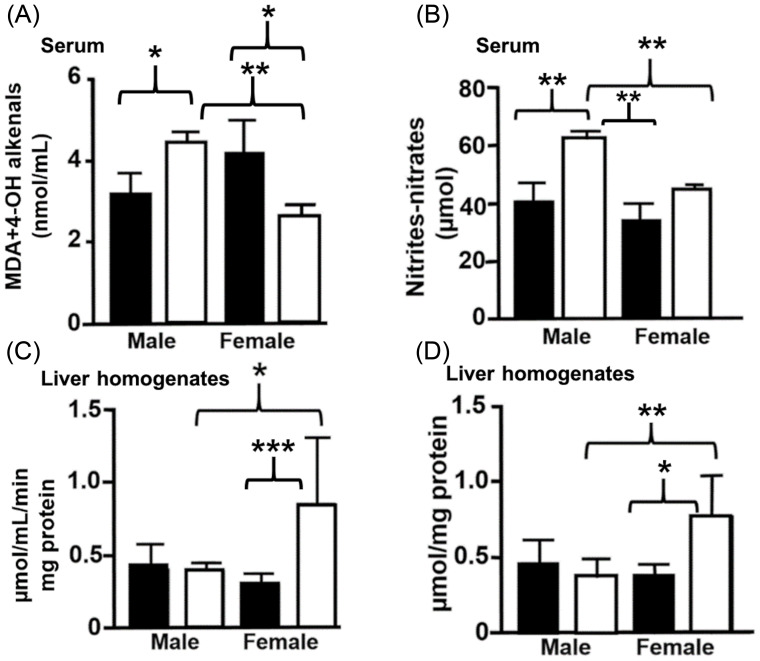
Oxidative stress markers in offspring of mothers fed with control and experimental (isocaloric low-protein diet) diets. Lipoperoxides (A) and nitrites-nitrates levels (B) were determined in serum; total antioxidant capacity (C), and catalase activity (D) in liver homogenates. Data are shown as mean ± standard error, ANOVA *P < 0.05, n = 4 rats per group.

Lipoperoxyde in serum levels in male offspring of mothers consuming the experimental diet were higher than those of male offspring of mothers consuming the control diet (P = 0.04). An increase in the experimental male offspring group was also observed, compared to the experimental female offspring (P = 0.01); experimental female offspring showed a decrease in lipoperoxyde levels in serum, compared to the control group (P = 0.003) (Fig. 5a). Serum nitrites-nitrates levels were higher in the experimental male offspring group, compared to the control male offspring group (P = 0.002). Serum levels of nitrites-nitrates showed an increase in the experimental male group, compared to the experimental female group (P = 0.001). A similar increase was observed in experimental male groups when compared to the control group in females (P = 0.001) (Fig. 5b). The antioxidant liver’s capacity decreased in the experimental male group, compared to the experimental female group (P = 0.02). Conversely, an increase in the liver’s antioxidant capacity was observed in experimental female group, compared to the control female group (P = 0.0005) (Fig. 5c). Catalase activity in the liver decreased in the experimental male group compared to the experimental female group (P = 0.0019) and increased in the experimental female group compared to the control female group (P = 0.01) (Fig. 5d).

### Breast milk and fat percentage/ percentage of total fatty acids

**Fig. 6.**
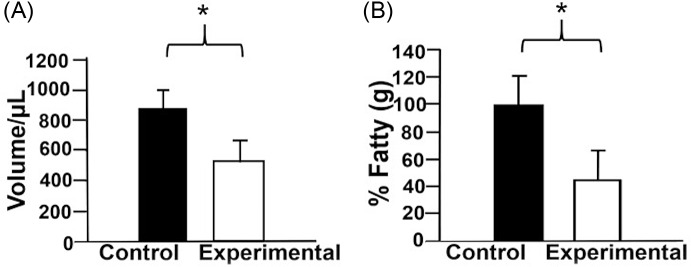
(A) Breast milk volume. All groups were fed either control diet (18% protein) or experimental diet (isocaloric low-protein diet, 6% protein). (B) Breast milk fat percentage: the dark bar for the control group, and the white bar for the experimental group. Data are shown as mean ± standard error. *P < 0.05, n = 3 rats per group.

It was observed that the milk volume of the experimental group was lower (533.33 µl) than that of the control group (866. 66 µl), corresponding to 34.5% of reduction (Fig. 6). Figure 6b shows the percentage of polyunsaturated fatty acids in breast milk from mothers exposed to the control and experimental diets with a 17.4% reduction (P < 0.05) in the experimental diet group compared to controls. The percentage of fatty acids (FA) in the milk of mothers fed with an experimental diet was 49.99%.

Table 2 shows the FA profile in both the experimental and control groups. The control group showed 2.23% of polyunsaturated fatty acid, while the ILPD group showed 1.29%. A significant decrease of 1.8% in polyunsaturated fatty acids in the breast milk of the experimental group was observed, when compared to the control group. In contrast, monounsaturated fatty acids showed a significant increase of 2% in the breast milk of mothers fed with the ILPD diet.

**Table 2.** Fatty acid profile in breast milk of mothers fed with control and experimental diets. Data are shown as mean ± standard error. *P < 0.05. n = 3 rats per group

Fatty acid	Control	Experimental	P value
*Butyric*	0.013 ± 0.03	0.14 ± 0.03	*0.51*
*Hexanoic*	0.16 ± 0.00	0.13 ± 0.01	*0.18*
*Octanoic*	3.69 ± 0.61	3.25 ± 0.42	*0.58*
*Nonanoic*	0.01 ± 0.00	0.01 ± 0.00	*0.48*
*Decanoic*	9.64 ± 0.26	9.23 ± 1.07	*0.73*
*Undecanoic*	0.01 ± 0.00	0.02 ± 0.00	*0.14*
*Dodecanoic*	5.47 ± 2.77	7.75 ± 1.37	*0.50*
*Tetradecanoic*	9.32 ± 2.00	8.03 ± 1.92	*0.66*
*Hexadecanoic*	21.09 ± 2.96	22.67 ± 1.09	*0.64*
*Octadecanoic*	4.07 ± 0.44	3.37 ± 0.34	*0.27*
***Docosanoic***	**0.10 ± 0.05**	**ND**	***0.03***
*Octadecadienoic*	0.07 ± 0.01	0.08 ± 0.00	*0.46*
*Palmitoleic acid*	0.15 ± 0.07	0.05 ± 0.05	*0.26*
*Palmitelaidic*	1.06 ± 0.34	2.17 ± 0.6	*0.18*
*Oleic*	15.16 ± 0.55	18.20 ± 2.5	*0.30*
*Elaidic*	1.66 ± 0.27	1.75 ± 0.33	*0.84*
*(Z)-10-Octadecenoic*	0.05 ± 0.01	0.02 ± 0.01	*0.36*
*Isopropyl oleate*	0.44 ± 0.06	0.37 ± 0.05	*0.48*
*Erucic*	0.04 ± 0.02	0.01 ± 0.00	*0.24*
*(E,E)- 9,12-octadecadienoic*	0.08 ± 0.00	0.09 ± 0.00	*0.06*
*(Z,Z)- 9,12-octadecadienoic*	18.31 ± 0.84	16.53 ± 0.33	*0.12*
***(Z,Z,Z)- 9,12,15-octadecatrienoic***	**2.23 ± 0.17**	**1.29 ± 0.08**	***0.00***
*Octadecatrienoic (isomer)*	0.13 ± 0.02	0.09 ± 0.01	*0.24*
*11,14-eicosadienoic*	1.06 ± 0.19	0.71 ± 0.05	*0.16*
*Eicosatrienoic isomer*	0.05 ± 0.00	0.05 ± 0.00	*0.91*
*7,10,13-eicosatrienoic acid*	0.76 ± 0.04	0.78 ± 0.06	*0.78*
*5,8,11,14-eicosatetraenoic acid*	2.24 ± 0.53	1.74 ± 0.32	*0.47*
*Eicosatetraenoic isomer*	0.07 ± 0.01	0.02 ± 0.01	*0.05*
*Eicosatetraenoic isomer*	0.11 ± 0.00	0.09 ± 0.00	*0.17*
*(9Z,12Z)-octadeca-9,12-dienoic*	0.06 ± 0.00	0.04 ± 0.00	*0.24*
*(5Z,8Z,11Z,14Z,17Z)-Icosa*- *5,8,11,14,17-Pentaenoic*	0.33 ± 0.04	0.26 ± 0.02	*0.27*
***Arachidonic***	**0.90 ± 0.35**	**ND**	***0.03***
*Docosahexaenoic*	0.5 ± 0.17	0.37 ± 0.11	*0.57*
*Saturated/polyunsaturated*	2.01	2.5	P *> 0.05*
*Saturated/monounsaturated*	2.77	2.41	**P** ***< 0.05***
Σ *saturated*	53.55	54.94	P *> 0.05*
Σ *monounsaturated*	19.2	22.7	**P** ***< 0.05***
Σ *polyunsaturated*	26.52	21.96	**P** ***< 0.05***

## Discussion

### Maternal food/water intake and body weight during early lactation

Adequate dietary intake before, during, and after pregnancy is essential for the proper development of the offspring’s central nervous system in the postnatal stage. Pregnancy is accompanied by significant changes in the maternal metabolism.^(32)^

Diet intake during pregnancy and lactation is associated with the fatty acid profile of breast milk.^(33)^ We observed a decrease from 15 to 20% in body weight in the experimental group during the evaluated days of lactation. In this regard, this productive period involves a higher metabolic demand for the mother compared to the pregnancy period. Breast milk production is satisfied by the mobilisation of the mother’s body tissues, as well as by increased food intake. Both processes cause alterations maternal body composition. In rodents, such adjustment depends partly on protein and energy intake.^(34,35)^ During lactation, the ILPD group showed weight loss. It has been described that the consumption of an ILPD could mobilise endogenous protein reserves through proteolysis in the maternal body to maintain the protein content of breast milk during lactation.^(7)^ Accordingly, it has been observed that ILPD consumption during gestation and lactation decreases the mother’s body weight from weaning in the postnatal stage.^(36)^ It’s known that DOHaD in early life environment can impact the risk of chronic diseases from childhood to adulthood and the mechanisms involved.^(6)^

Breast milk influences the development and functioning of the offspring’s nervous system, including the hippocampus; it is known that the consumption of an ILPD, high-carbohydrate diet is related to modifications in the structure and function of memory and learning, including functional changes in synaptic communication such as synaptogenesis.^(37)^ However, it is unknown whether ILPD intake during pregnancy and lactation affects the fatty acid profile in breast milk^(38)^ and the hippocampal synaptogenesis in the early postnatal stage, as well as whether it promotes changes in oxidative stress markers in offspring during the postnatal period. If a nutritional deficit occurs, the maternal environment faces a metabolic and physiological challenge in order to ensure proper development of the gestational product.^(2)^ Food intake observed during lactation showed a tendency to decrease in the ILPD group, which is consistent with another research.^(39)^ Research based on experimental designs has shown that maternal body weight tends to decrease during lactation and that the offspring tend to have a lower birth weight when fed with low-protein diets,^(40)^ which is consistent with the findings of this study.

### Synaptophysin area ratio in the hippocampus of offspring at PND10

The decreased Syn area ratio in the hippocampus of male offspring whose mothers consumed an ILPD is consistent with previous studies, where adult male offspring of high-fat-fed mothers showed impaired object and spatial recognition memory.^(39,41,42)^ The hippocampal CA1 region is one of the most evolutionarily conserved cortical regions involved in memory consolidation and retrieval.^(43)^ Pyramidal cell axons constitute the main source of synapses in hippocampal formation, which is essential for spatial navigation and episodic memory.^(44)^ There are sex differences in brain areas, such as the formation of the hippocampus.^(45)^ It is important to observe the sexual dimorphism related to synaptic function during early postnatal development. Our results suggest a greater number of synapses in the hippocampus of female offspring in the postnatal stage (whose mothers were fed with the ILPD), since their Syn area ratio was significantly higher than in males. It is suggested that this result is associated with malnutrition, since the protein deficit reduces Syn in these analysed regions of the male hippocampus. At this postnatal age, males may be more vulnerable to the mother’s diet during gestation and lactation.

The CA3 region of the hippocampus is involved in information processing and encoding of short-term memory, as well as in retrieval of long-term memory.^(46)^ Experimental evidence indicates a decrease in the Syn area ratio in CA3 in the postnatal stage due to the consumption of poor diets.^(47,48)^ It is suggested that males’ hippocampus could be more sensitive to diet characteristics, yet we continue to reflect upon other proper explanations. In support of this, it has been described that the consumption of an ILPD during pregnancy affects neuronal cells in the CA3 region in late postnatal stages,^(49)^ and that it induces a deficit in short-term memory in late postnatal periods.^(50)^ Notwithstanding, those studies did not differentiate by sex. On the other hand, it has been reported that the offspring of mothers with gestational diabetes showed a decrease in Syn in the CA3 region cells, suggesting that hyperglycaemia is another factor that could alter the structure and function of hippocampal development.^(51)^ Studies with PUFA supplementation have shown an overexpression of Syn in the hippocampus, suggesting an increase in the connectivity of these neurons.^(52)^ It has also been observed that dietary proline and glycine supplementation have a positive effect on Syn expression in the hippocampal CA3 region in adulthood.^(53)^ It has been reported that a decrease in Syn expression is associated with spatial learning and memory deficits in older age,^(54)^ and it is proposed that low-protein diets may have negative effects on synaptogenesis processing in hippocampal regions.^(55)^ Neurogenesis is gradual, the pyramidal neurons in CA1 are generated from E16 to E21.^(56,57)^ As the hippocampal neurons develop, they generate synapses and circuits within the entire hippocampal network and the different brain areas. Our results are consistent with previous studies that reported alterations in the hippocampal cytoarchitecture, such as a decrease in the pyramidal and granular neurons,^(58,59)^ a decrease in the cortical thickness and the dendritic spines density,^(60,61)^ as well as neurotransmission system alterations.^(62)^ In this study, the Syn was evaluated in the hippocampus at PND10; it is relevant to mention that the results of our study demonstrate that these structural plastic changes in synapses occur from an early stage at PND10.

The hippocampus is an essential brain structure involved in information processing related to learning and memory.^(63,64)^

The input of information from the entorhinal cortex to the *radiatum* and *oriens* stratum of CA1 occurs from E15, while in the molecular stratum of DG it occurs from E18 and E19.^(65)^ These results are relevant and could reflect an alteration in the connectivity observed in PND10 that directly affects the cognitive performance of juvenile and adult offspring, as previously reported.^(41,66)^ These results demonstrate that synaptogenesis perturbation observed in PND10 could contribute to cognitive and behavioural deficits. Finally, another relevant finding is that the observed effect on the proportion of the area occupied by Syn in the dorsal hippocampus occurred exclusively in male offspring. The lack of effect on female offspring could be due to the protective role of sex hormones in the neurodevelopment of offspring. It has been reported that high levels of oestradiol in the mother during pregnancy and lactation contribute to mitigate the detrimental effects on the offspring’s heart, by decreasing oxidative stress markers, and increasing antioxidant enzymatic activity.^(67)^ The brain also exhibits sex-specific gene expression patterns and epigenetic changes, underlining the significance of sex chromosomes in brain function and growth.^(68)^

### Pro-oxidative markers in offspring

Studies have shown that a maternal ILPD increases postnatal susceptibility to the development of metabolic diseases during adulthood.^(69)^ High levels of oestrogens during reproductive age may promote resistance to oxidative damage in the brainstem, a fact that is evident in prepubertal rats.^(70)^ We showed that experimental male pups presented higher levels of nitrites and nitrates in serum when compared with the experimental females, and the levels of catalase and antioxidant capacity in the liver decreased when compared with the experimental females, which could mean that males are more vulnerable.

### Breast milk and lipidic content

In particular, findings regarding the breast milk production of mothers fed with an ILPD during lactation suggest that milk’s fat content could be an adaptative mechanism.^(38)^ Our study showed a decrease in milk’s fat content in the ILPD group, as well as a lower amount of polyunsaturated and monounsaturated fatty acids, which is consistent with another research.^(71)^ Milk volume also decreased in mothers fed with an ILPD. Studies have shown that fatty acids in breast milk have an important role in the development of the offspring’s nervous system in the postnatal stage,^(69)^ especially the polyunsaturated fatty acids (PUFA), which are crucial structural components of cell membranes that play a key role in neurological development.^(13)^

Our results show decreased concentrations of arachidonic acid (AA), α-linolenic acid (ALA), and eicosanoic acid. Omega-6 and Omega-9 fatty acids are considered essential and, therefore, it is necessary to include them in proper proportions in the offspring’s diet, since an imbalance or their absence causes metabolic and neurological alterations in the postnatal stage.^(72)^ It is suggested that this effect may be related to a process of assimilation, not to availability. It has been found that AA and ALA are essential in the metabolism, maturation, and establishment of the brain structure.^(73)^ It has been reported that a chronic polyunsaturated fatty acids deficiency may contribute to the development of disorders, such as attention deficit hyperactivity disorder in childhood. On the other hand, monounsaturated fatty acids (MUFAs) are emerging health biomarkers; in particular, the ratio of palmitoleic acid (9cis-16:1) to palmitic acid (16:0) provides the delta-9 desaturase ratio, which increases metabolic disorders.^(74)^ Interestingly, we found that the palmitic/palmitoleic ratio in the control group is 141.5, while in the ILPD group it is 390. Consistently, a high-carbohydrate diet promotes lipid deposition and inflammation in the brain of mice,^(75)^ which is associated with oxidative stress. Protein restriction, and high carbohydrate intake during pregnancy not only affects the maternal metabolism, but also the offspring’s metabolism; the mobilisation of maternal reserves may be insufficient to provide adequate nutrients.^(76)^ In conclusion, the results of this research suggest that the reduction of the hippocampal Syn area ratio, as well as the increase of pro-oxidant state in serum and liver of the offspring at PND10, exclusively affect males, which could be more vulnerable to the mother’s consumption of an ILPD during pregnancy.

### Limitations of the study

Given that the mother is the biological replicate, an acknowledged limitation of the present study is the low litter numbers used and thus is representative of a pilot study.

## Supporting information

Bello-Medina et al. supplementary material Bello-Medina et al. supplementary material

## Data Availability

Authors confirm that the data supporting the findings of this study are available. This submission has been approved by all co-authors.

## Abbreviations

**AA:** arachidonic acid; **ADHD:** deficit hyperactivity disorder; **ALA:** α-linolenic acid; **DOHaD:** developmental origins of health and disease; **EI:** Electron Impact; **FAMES:** Fatty Acids Methyl Esters; **HPLC:** high-performance liquid chromatography; **ILPD:** Low-Protein Diet; **MUFA:** monounsaturated fatty acids; **PND10:** Postnatal day 10; **PUFA:** polyunsaturated fatty acids; **Syn:** Synaptophysin.

## Supplementary material

The supplementary material for this article can be found at https://doi.org/10.1017/jns.2024.46
