# Supplementary material for: A maternal low-protein diet results in sex-specific differences in synaptophysin expression and milk fatty acid profiles in neonatal rats
Source: J Nutr Sci. 2024 Oct 14;13:e64. doi: 10.1017/jns.2024.46 (PMC11514622; doi:10.1017/jns.2024.46)
Supplement: Bello-Medina et al. supplementary material [file S2048679024000466sup001.pdf]

Multivariate Analysis

				value	F	hypothesis's df	degree of error	sig
Control	Female	Intersection	Pillai's trace statistics	0.200	.250[b]	1.000	1.000	0.705
			Wilks's lambda distribution	0.800	.250[b]	1.000	1.000	0.705
			Hotelling Trace	0.250	.250[b]	1.000	1.000	0.705
			Roy's largest root	0.250	.250[b]	1.000	1.000	0.705
		Group * sex * weight at PND10	Pillai's trace statistics	0.091	.101[b]	1.000	1.000	0.804
			Wilks's lambda distribution	0.909	.101[b]	1.000	1.000	0.804
			Hotelling Trace	0.101	.101[b]	1.000	1.000	0.804
			Roy's largest root	0.101	.101[b]	1.000	1.000	0.804
	Male	Intersection	Pillai's trace statistics	0.820	4.546[b]	1.000	1.000	0.279
			Wilks's lambda distribution	0.180	4.546[b]	1.000	1.000	0.279
			Hotelling Trace	4.546	4.546[b]	1.000	1.000	0.279
			Roy's largest root	4.546	4.546[b]	1.000	1.000	0.279
		Group * sex * weight at PND10	Pillai's trace statistics	0.760	3.175[b]	1.000	1.000	0.326
			Wilks's lambda distribution	0.240	3.175[b]	1.000	1.000	0.326
			Hotelling Trace	3.175	3.175[b]	1.000	1.000	0.326
			Roy's largest root	3.175	3.175[b]	1.000	1.000	0.326
Experimental	Female	Intersection	Pillai's trace statistics	0.918	11.177[b]	1.000	1.000	0.185
			Wilks's lambda distribution	0.082	11.177[b]	1.000	1.000	0.185
			Hotelling Trace	11.177	11.177[b]	1.000	1.000	0.185
			Roy's largest root	11.177	11.177[b]	1.000	1.000	0.185
		Group * sex * weight at PND10	Pillai's trace statistics	0.761	3.189[b]	1.000	1.000	0.325
			Wilks's lambda distribution	0.239	3.189[b]	1.000	1.000	0.325
			Hotelling Trace	3.189	3.189[b]	1.000	1.000	0.325
			Roy's largest root	3.189	3.189[b]	1.000	1.000	0.325
	Male	Intersection	Pillai's trace statistics	0.186	.229[b]	1.000	1.000	0.716
			Wilks's lambda distribution	0.814	.229[b]	1.000	1.000	0.716
			Hotelling Trace	0.229	.229[b]	1.000	1.000	0.716
			Roy's largest root	0.229	.229[b]	1.000	1.000	0.716
		Group * sex * weight at PND10	Pillai's trace statistics	0.951	19.253[b]	1.000	1.000	0.143
			Wilks's lambda distribution	0.049	19.253[b]	1.000	1.000	0.143
			Hotelling Trace	19.253	19.253[b]	1.000	1.000	0.143
			Roy's largest root	19.253	19.253[b]	1.000	1.000	0.143
